# Positive association between baseline brachial–ankle pulse wave velocity and the risk of new-onset diabetes in hypertensive patients

**DOI:** 10.1186/s12933-019-0915-0

**Published:** 2019-08-28

**Authors:** Yuanyuan Zhang, Panpan He, Youbao Li, Yan Zhang, Jianping Li, Min Liang, Guobao Wang, Genfu Tang, Yun Song, Binyan Wang, Chengzhang Liu, Lishun Liu, Yimin Cui, Xiaobin Wang, Yong Huo, Xiping Xu, Xianhui Qin

**Affiliations:** 1 National Clinical Research Center for Kidney Disease; The State Key Laboratory for Organ Failure Research; Division of Nephrology, Nanfang Hospital, Southern Medical University, Guangzhou, 510515 China; 2 Department of Cardiology, Peking University First Hospital, Beijing, 100034 China; 3 Health Management College, Anhui Medical University, Hefei, 230032 China; 4 Beijing Advanced Innovation Center for Food Nutrition and Human Health, College of Food Science and Nutritional Engineering, China Agricultural University, Beijing, 100083 China; 5 Institute of Biomedicine, Anhui Medical University, Hefei, 230032 China; 6 Department of Pharmacy, Peking University First Hospital, Beijing, 100034 China; 7 Department of Population, Family and Reproductive Health, Johns Hopkins University Bloomberg School of Public Health, 615 N. Wolfe Street, E4132, Baltimore, MD 21205-2179 USA

**Keywords:** Brachial–ankle pulse wave velocity, New-onset diabetes, Fasting glucose, Hypertensive patients

## Abstract

**Background:**

There is no clearly defined temporal relationship between arterial stiffness and diabetes. We aimed to investigate the prospective association between baseline brachial–ankle pulse wave velocity (baPWV) and the risk of new-onset diabetes during follow-up, and examined whether there were effect modifiers, in hypertensive patients.

**Methods:**

We included 2429 hypertensive patients with all the pertinent data but without diabetes at the baseline, who were part of the China Stroke Primary Prevention Trial (CSPPT), a randomized, double-blind, actively controlled trial conducted in 32 communities in Anhui and Jiangsu provinces in China. The primary outcome was new-onset diabetes, defined as physician-diagnosed diabetes or use of glucose-lowering drugs during follow-up, or fasting glucose (FG) ≥ 126.0 mg/dL at the exit visit.

**Results:**

During a median follow-up duration of 4.5 years, 287 (11.8%) participants developed diabetes. There was a significant positive association between baseline baPWV and the risk of new-onset diabetes (per SD increment; OR, 1.33; 95% CI 1.13, 1.56). Consistently, when baPWV was assessed as quartiles, a significantly higher risk of new-onset diabetes was found in participants in quartiles 2–4 (≥ 15.9 m/s; OR, 1.80; 95% CI 1.22, 2.65) compared with those in quartile 1 (< 15.9 m/s). The positive association was consistent in participants with (per SD increment; OR, 1.29; 95% CI 1.06, 1.56) or without (per SD increment; OR, 1.40; 95% CI 1.15, 1.71) impaired fasting glucose (IFG, FG ≥ 100.8 and < 126.0 mg/dL, *P*-interaction = 0.486).

**Conclusions:**

In this sample of hypertensive patients, we found a significant positive association between baseline baPWV and the risk of new-onset diabetes.

*Clinical trial registration* Trial registration: NCT00794885 (clinicaltrials.gov). Retrospectively registered November 20, 2008

## Background

Diabetes is a worldwide public health problem [1]. Diabetes has been reported to be an important risk factor for cardiovascular disease (CVD) [2] and chronic kidney diseases [3]. Therefore, early identification of individuals at high-risk of developing diabetes is of clinical importance, for early risk assessment and intervention can help prevent the onset and slow the progress of diabetes and its related CVD complications.

Arterial stiffness increases with advancing age and has been regarded as an important risk factor for age-related morbidity and mortality [4, 5]. Currently, because of its reproducibility and simplicity [6, 7], brachia–ankle pulse wave velocity (baPWV) is a widely used method for assessing arterial stiffness in studies of large sample sizes [7]. baPWV was found to be related to increased risk of stroke [8], total cardiovascular events and all-cause mortality [9], and was shown to positively associated with hypertension [10] and the presence of coronary calcium [11] or left ventricular mass [12]. Nevertheless, there are limited studies on baPWV and diabetes. Only some cross-sectional studies [13–15] reported that baPWV was related to the prevalence of diabetes.

Of note, we have previously demonstrated that higher baPWV decreased the antihypertensive effect of antihypertensive treatment in hypertensive adults [16]. Another study showed that uncontrolled blood pressure (BP) was associated with increased risk of incident diabetes [17]. These results raise the possibility that there is a link between baPWV and incident diabetes, but few studies have been conducted to delineate their temporal and causal relationships [18].

To address the aforementioned gap, the current study aimed to evaluate the prospective association between baseline baPWV and new-onset diabetes during the longitudinal follow-up, and to examine any possible effect modifiers among hypertensive patients, using data from the China Stroke Primary Prevention Trial (CSPPT) [19, 20].

## Methods

### Study participants and design

All participants were part of the CSPPT (clinicaltrials.gov identifier: NCT00794885). Detailed methods and major findings of the CSPPT trial have been described previously [19–22]. Briefly, the CSPPT was a large, community-based, multi-site, randomized, double-blind, and actively controlled trial with a total of 20,702 participants in China. Eligible participants were men and women aged 45 to 75 years old who had hypertension, defined as seated, resting systolic BP (SBP) ≥ 140 mmHg or diastolic BP (DBP) ≥ 90 mmHg at both the screening and the recruitment visit, or who were on antihypertensive medications. The major exclusion criteria included history of physician-diagnosed stroke, myocardial infarction, heart failure, post-coronary revascularization, and/or congenital heart disease.

The present study is a post hoc analysis of the CSPPT on 3532 subjects with baPWV measurements at baseline. Of those, 2429 participants without peripheral artery occlusive disease (PAD) assessed by ankle–brachial index (ABI < 0.9) [6, 16], with complete data on fasting glucose at baseline, and with physician-diagnosed diabetes or use of glucose-lowering drugs during the follow-up or fasting glucose data at the exit visit, as well as who were free of diabetes (physician-diagnosed diabetes or using glucose-lowering drugs) and whose fasting glucose (FG) was < 126.0 mg/dL at baseline, were included in the final analysis (Additional file 1: Figure S1).

The parent study (the CSPPT) and the current study were approved by the Ethics Committee of the Institute of Biomedicine, Anhui Medical University, Hefei, China (Federalwide Assurance Number 00001263). All participants provided written informed consent.

### Intervention and follow-up

Eligible participants were randomly assigned, in a 1:1 ratio, to one of two treatment groups: a daily oral dose of one tablet containing 10 mg enalapril and 0.8 mg folic acid (the enalapril–folic acid group), or a daily oral dose of one tablet containing 10 mg enalapril only (the enalapril group). Participants were followed up every 3 months. During each follow-up visit, blood pressure was measured; study drug compliance, concomitant medication use, adverse events and possible endpoint events were documented by trained research staff and physicians. The study drug compliance was calculated as the percentage of days taking the study drugs during the trial.

### Data collection procedures

Baseline data collection was conducted by trained research staff according to a standard operating procedure. Each participant was interviewed using a standardized questionnaire designed specifically for this study. The question about socioeconomic status was phrased as follows, “How does your standard of living compare to others?” and a choice of three responses: bad, medium, and good was provided. The question about physical activity was phrased as follows, “How do you describe your daily physical activity level?” and a choice of three responses: low, moderate, and high was provided [23–25].

### Laboratory assays

Serum fasting glucose (FG), lipids, and creatinine levels were measured using automatic clinical analyzers (Beckman Coulter) at the core laboratory of the National Clinical Research Center for Kidney Disease, Nanfang Hospital, Guangzhou, China. Serum folate was measured at baseline by a commercial laboratory using a chemiluminescent immunoassay (New Industrial).

### baPWV measurements

baPWV, calculated as the ratio of transmission distance from the brachium to the ankle divided by the transit time, was used in this study. Participants were asked to remain in the supine position for at least 5 min after which baseline baPWV was measured using an automatic waveform analyzer (form PWV/ABI, BP-203RPE; Omron-Colin, Japan) according to published guidelines. The details describing the method of obtaining baPWV measurements are published elsewhere [8, 16]. In brief, two, bilateral readings of baPWV measurements were simultaneously taken and the maximum reading from each side was used for the analysis.

### Study outcomes

The primary study outcome was new-onset diabetes, defined as physician-diagnosed diabetes, or use of glucose-lowering drugs during follow-up, or new onset FG ≥ 126.0 mg/dL at the exit visit.

The secondary study outcomes include: (1) physician-diagnosed diabetes, or use of glucose-lowering drugs during follow-up; (2) the change in FG, calculated as FG at the exit visit minus that at baseline. The analysis of change in FG included subjects without physician-diagnosed diabetes, or use of glucose-lowering drugs during the follow-up.

### Statistical analyses

Baseline characteristics are presented as mean ± standard deviation (SD) for continuous variables and proportions for categorical variables. Differences in baseline characteristics by baPWV quartiles were compared using ANOVA tests, or Chi-square tests, accordingly. The relationship of baPWV quartiles (< 15.9, 15.9–< 17.9, 17.9–< 20.7, and ≥ 20.7 m/s) with new-onset diabetes (primary outcome), physician-diagnosed diabetes or use of glucose-lowering drugs during follow-up, and change in FG (secondary outcomes) were evaluated using multivariable logistic regression models, Cox proportional hazard regression models and generalized linear regression models, respectively, without and with adjustment for age, sex, study center, study treatment group, body mass index (BMI), heart rate, smoking, systolic blood pressure (SBP), fasting glucose, total cholesterol (TC), creatinine, and folate at baseline, as well as time-averaged SBP during the treatment period. As additional exploratory analyses, possible modifications on the association between baPWV and new-onset diabetes were also evaluated by stratified analyses and interaction testing.

A two-tailed *P *< 0.05 was considered to be statistically significant in all analyses. R software (version 3.4.3, http://www.R-project.org) were used for all statistical analyses.

## Results

### Study participants and baseline characteristics

As illustrated in the flow chart (Additional file 1: Figure S1), a total of 2429 hypertensive participants of the CSPPT without diabetes at the baseline were included in the final analysis.

Baseline characteristics of the study participants by baPWV quartiles are shown in Table 1. The mean age of the participants was 59.7 (SD, 7.4) years; 1049 were men (43.2%). Mean baseline baPWV was 18.6 (SD, 3.7) m/s. baPWV levels were inversely associated with BMI and time averaged diastolic blood pressure (DBP) during the treatment period, and positively associated with age, heart rate, SBP, DBP, TC, fasting glucose, high-density-lipid cholesterol (HDL-C) at baseline, as well as time averaged SBP during the treatment period. Moreover, participants with higher baPWV seemed to have lower physical activity levels (Additional file 1: Table S1).

**Table 1:** Baseline characteristics of the study population by brachial–ankle pulse wave velocity (baPWV) quartiles

Variables	Total	baPWV, m/s				*P* value
		Quartile 1 (< 15.9)	Quartile 2 (15.9–< 17.9)	Quartile 3 (17.9–< 20.7)	Quartile 4 (≥ 20.7)	
N	2429	607	605	606	611	
Age, years	59.7 ± 7.4	55.2 ± 6.3	58.9 ± 6.8	60.9 ± 6.9	63.8 ± 6.6	< 0.001
Male, No. (%)	1049 (43.2)	273 (45.0)	267 (44.1)	252 (41.6)	257 (42.1)	0.581
Body mass index, kg/m^2^	25.1 ± 3.6	25.7 ± 3.5	25.2 ± 3.7	24.8 ± 3.5	24.5 ± 3.4	< 0.001
Heart rate, bmp	72.4 ± 9.5	70.1 ± 8.5	71.2 ± 8.5	72.3 ± 9.2	75.9 ± 10.7	< 0.001
baPWV, m/s	18.6 ± 3.7	14.6 ± 1.1	16.9 ± 0.6	19.2 ± 0.8	23.7 ± 2.8	< 0.001
Current smoking, No. (%)	585 (24.1)	127 (20.9)	155 (25.6)	151 (24.9)	152 (24.9)	0.275
Enalapril group, No. (%)	1196 (49.2)	304 (50.1)	290 (47.9)	295 (48.7)	307 (50.2)	0.823
*MTHFR* C677T polymorphisms, No. (%)						0.920
CC	636 (26.2)	151 (24.9)	156 (25.8)	169 (27.9)	160 (26.2)	
CT	1186 (48.8)	296 (48.8)	297 (49.1)	293 (48.3)	300 (49.1)	
TT	607 (25.0)	160 (26.4)	152 (25.1)	144 (23.8)	151 (24.7)	
BP, mmHg						
Systolic BP at baseline	166.5 ± 20.2	153.5 ± 16.2	163.4 ± 16.1	170.0 ± 18.8	179.1 ± 20.0	< 0.001
Diastolic BP at baseline	94.8 ± 11.7	93.2 ± 10.3	94.1 ± 11.3	94.5 ± 12.0	97.4 ± 12.6	< 0.001
Time-averaged systolic BP^a^	138.6 ± 10.7	133.7 ± 9.0	137.8 ± 9.9	139.2 ± 10.1	143.5 ± 11.2	< 0.001
Time-averaged diastolic BP	83.3 ± 7.4	84.6 ± 6.8	83.6 ± 7.4	82.5 ± 7.6	82.4 ± 7.4	< 0.001
Laboratory results						
HDL-C, mmol/L	1.3 ± 0.3	1.3 ± 0.3	1.3 ± 0.4	1.4 ± 0.3	1.4 ± 0.4	< 0.001
Total cholesterol, mmol/L	5.4 ± 1.1	5.3 ± 1.0	5.4 ± 1.1	5.5 ± 1.1	5.6 ± 1.2	< 0.001
Triglycerides, mmol/L	1.7 ± 2.1	1.6 ± 1.0	1.7 ± 4.0	1.6 ± 0.8	1.7 ± 0.9	0.606
Fasting glucose, mg/dL	97.6 ± 12.5	97.0 ± 11.9	97.3 ± 11.9	97.1 ± 12.7	99.0 ± 13.2	0.016
Creatinine, μmol/L	65.3 ± 15.2	64.6 ± 14.1	65.2 ± 14.1	65.8 ± 17.3	65.7 ± 15.2	0.466
Folate, ng/mL	8.2 ± 3.6	8.0 ± 3.3	8.2 ± 3.5	8.1 ± 3.6	8.4 ± 3.9	0.213
Medication use, No. (%)						
Antihypertensive drugs	1152 (47.4)	292 (48.1)	276 (45.6)	294 (48.5)	290 (47.5)	0.755
Lipid lowering drugs	18 (0.7)	6 (1.0)	7 (1.2)	1 (0.2)	4 (0.7)	0.193
Antiplatelet drugs	67 (2.8)	17 (2.8)	12 (2.0)	14 (2.3)	24 (3.9)	0.177

Variables are presented as mean ± SD or n (%)

BP: blood pressure; HDL-C: high-density lipoprotein; *MTHFR*: methylenetetrahydrofolate reductase

^a^Time-averaged systolic BP: mean systolic BP during the treatment period

### Association between baseline baPWV and the study outcomes

During a median follow-up duration of 4.5 years (IQR, 4.2–4.7 years), new-onset diabetes occurred in 287 (11.8%) participants.

**Fig. 1:**
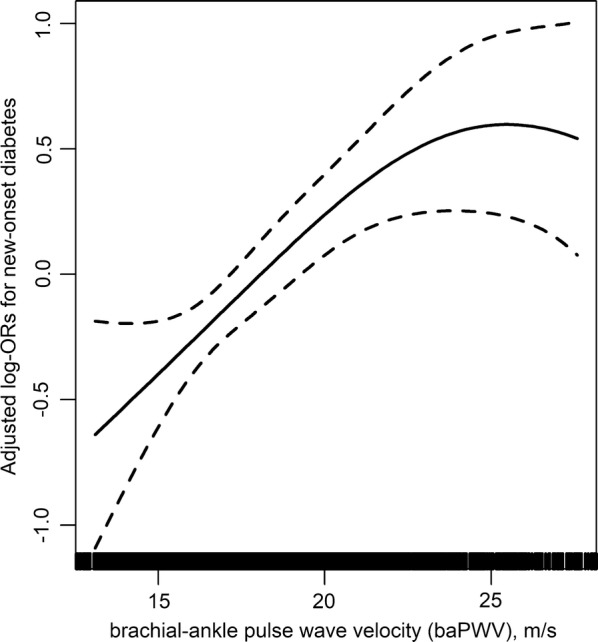
Association between baseline brachial–ankle pulse wave velocity (baPWV) and new-onset diabetes during follow-up. *Adjusted for age, sex, study center, study treatment group, body mass index (BMI), heart rate, smoking, systolic blood pressure (SBP), fasting glucose (FG), total cholesterol (TC), creatinine, and folate at baseline, as well as time-averaged SBP during the treatment period

**Fig. 2:**
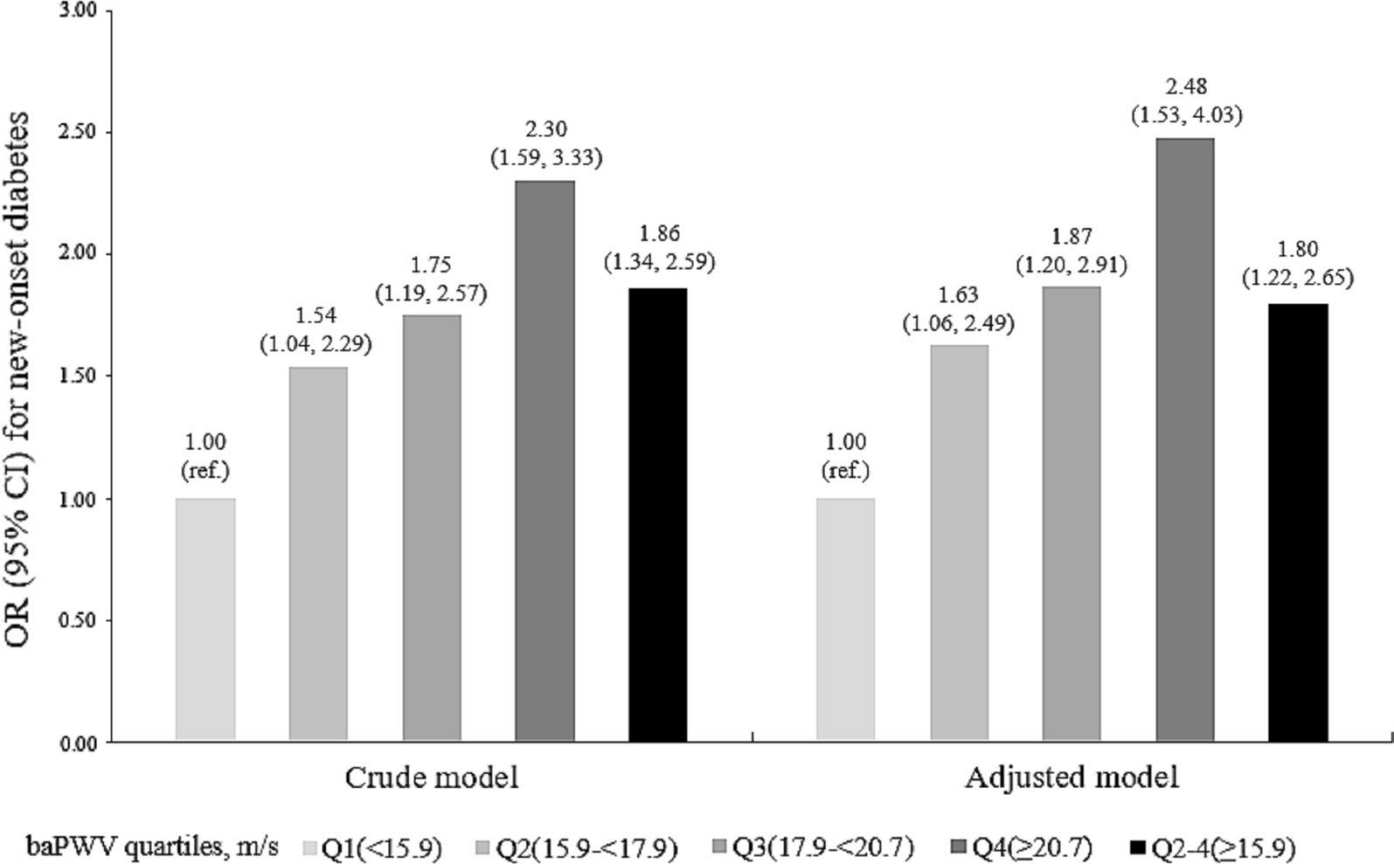
Risk of new-onset diabetes (expressed as OR and 95% CI) based on brachial–ankle pulse wave velocity (baPWV) quartiles. *Adjusted for age, sex, study center, study treatment group, body mass index (BMI), heart rate, smoking, systolic blood pressure (SBP), fasting glucose (FG), total cholesterol (TC), creatinine, and folate at baseline, as well as time-averaged SBP during the treatment period

Overall, there was a significant positive association between baPWV and the risk of new-onset diabetes (Fig. 1). Per SD increment (3.7 m/s), higher baPWV was associated with a 33% increase in the adjusted risk of new-onset diabetes (OR, 1.33; 95% CI 1.13, 1.56) (Additional file 1: Table S2). When baPWV was assessed as quartiles, the adjusted odds ratios for participants in the second, third and fourth quartiles were 1.63 (95% CI 1.06, 2.49), 1.87 (95% CI 1.20, 2.91) and 2.48 (95% CI 1.53, 4.03), respectively, compared with those in quartile 1 (*P* for trend < 0.001). A significantly higher risk of new-onset diabetes was found in participants in quartiles 2–4 (adjusted OR, 1.80; 95% CI 1.22, 2.65) compared with those in quartile 1 (Fig. 2, Additional file 1: Table S2).

Consistently, per each SD increment (3.7 m/s), higher baPWV was associated with a 61% increase in the adjusted risk of physician-diagnosed diabetes or use of glucose-lowering drugs during follow-up (HR, 1.61; 95% CI 1.05, 2.47) (Additional file 1: Figure S2, Table S3). Accordingly, a significantly higher increase of FG levels (FG at the exit visit minus that at baseline) was also found in participants in quartiles 4 (adjusted β, 2.89 mg/dL; 95% CI 0.12, 5.66) compared with those in quartile 1 of the baPWV levels among participants without physician-diagnosed diabetes, or use of glucose-lowering drugs during follow-up (Additional file 1: Figure S3, Table S4).

### Sensitivity analysis

During the treatment period, participants with higher baPWV had a higher frequency in use of calcium channel blockers (CCB) or diuretics (Additional file 1: Table S5). However, further adjustment for two variables: the use of calcium channel blockers (CCB), and the use of diuretics during the treatment period, did not substantially change the association between baPWV and new-onset diabetes (Additional file 1: Table S6). Moreover, the similar results were also found in participants without the concomitant use of diuretics during the treatment period (Additional file 1: Table S7).

In the CSPPT, all participants used enalapril or enalapril- folic acid during the follow up. However, further adjustment for the study drug (enalapril or enalapril-folic acid) compliance during the trial did not substantially change the results (Additional file 1: Table S8). Furthermore, we have further adjusted for family history of diabetes, physical activity, alcohol consumption and socioeconomic status, the association between baPWV and new-onset diabetes also did not been changed materially (Additional file 1: Table S9). More importantly, the similar results were also observed with further adjustment for the change in BMI (calculated as BMI at the exit visit minus that at baseline) (Additional file 1: Table S10).

In addition, we further explored the relationship of pulse pressure (PP) (both baseline PP and time-averaged PP during the treatment period) with new-onset diabetes (Additional file 1: Table S11). Overall, there was no significant association between baseline PP and new-onset diabetes. However, there was a significant positive relationship of time-averaged PP with new-onset diabetes (quartile 3–4 vs. quartile 1; adjusted OR, 1.55; 95% CI 1.03, 2.33). Nevertheless, further adjustment for baseline PP and time-averaged PP during the treatment period did not substantially alter the positive association between baseline baPWV and the risk of new-onset diabetes (per SD increment; adjusted OR, 1.30; 95% CI 1.11, 1.61) (Additional file 1: Table S12).

### Subgroup analyses by potential effect modifiers

**Fig. 3:**
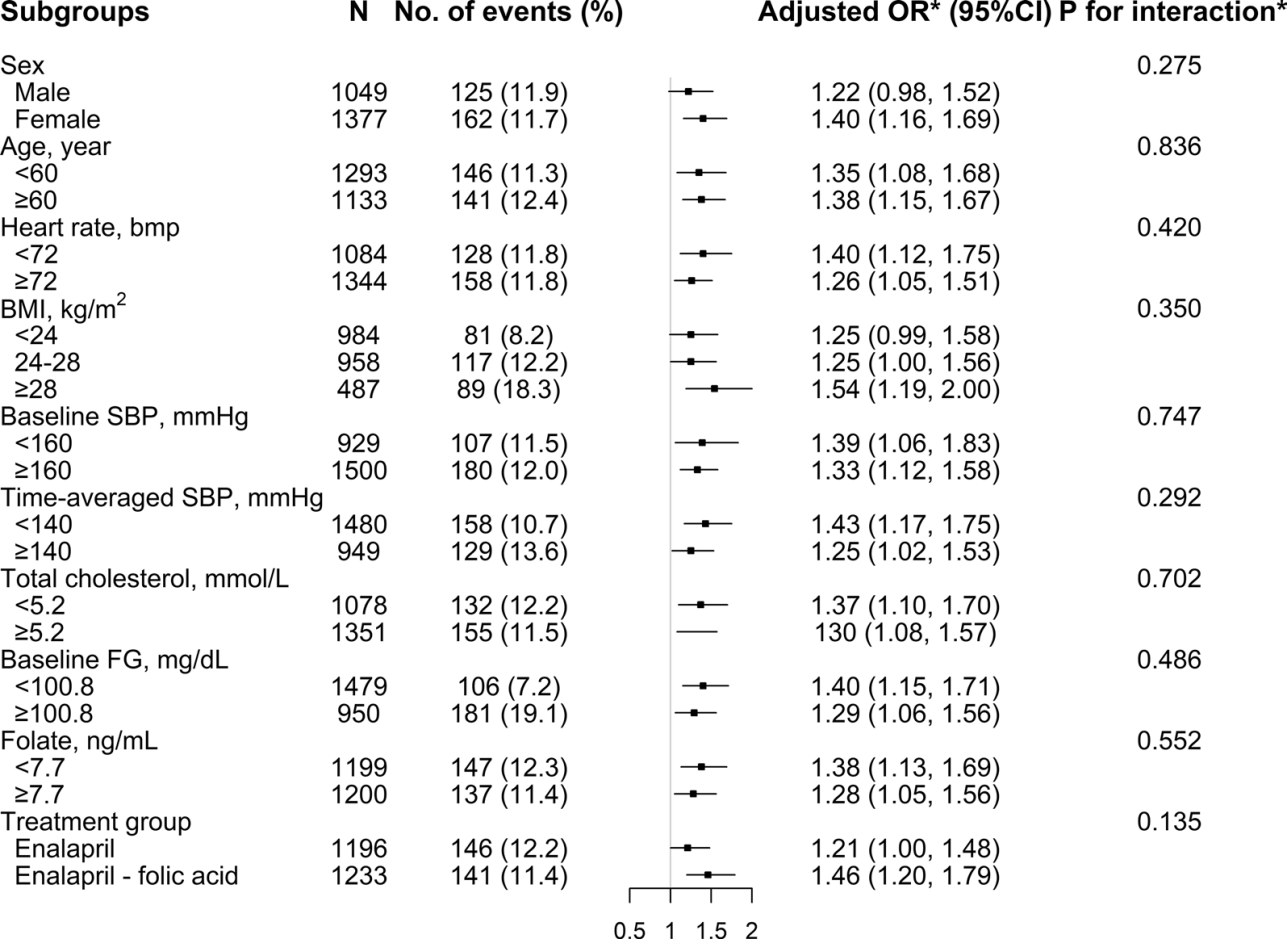
Association between baseline brachial–ankle pulse wave velocity (baPWV) (per SD increment) and new-onset diabetes during follow-up in various subgroups. *Adjusted for age, sex, study center, study treatment group, body mass index (BMI), heart rate, smoking, systolic blood pressure (SBP), fasting glucose (FG), total cholesterol (TC), creatinine, and folate at baseline, as well as time-averaged SBP during the treatment period, if not be stratified

None of other variables, including sex (*P*-interaction = 0.275), age (< 60 vs. ≥ 60 years; *P*-interaction = 0.836), BMI (< 24, 24–28, ≥ 28 kg/m^2^; *P*-interaction = 0.350), treatment group (enalapril vs. enalapril + folic acid; *P*-interaction = 0.135), heart rate [< 72 (median) vs. ≥ 72 bmp; *P*-interaction = 0.420], SBP (< 160 vs. ≥ 160 mmHg; *P*-interaction = 0.747), TC [< 5.2 vs. ≥ 5.2 mmol/L; *P*-interaction = 0.702], FG (< 100.8 vs. 100.8–< 126 mg/mL; *P*-interaction = 0.486), folate [< 7.7 (median) vs. ≥ 7.7 ng/mL; *P*-interaction = 0.552] at baseline, and time-averaged SBP (< 140 vs. ≥ 140 mmHg; *P*-interaction = 0.292) during the treatment period, significantly modified the association between baPWV and new-onset diabetes (Fig. 3).

## Discussion

To our knowledge, this is the first prospective study to demonstrate that baseline baPWV was positively associated with the risk of new-onset diabetes during a median follow-up of 4.5 years in hypertensive patients. The positive association was consistent across the strata for treatment group, sex, age, BMI, heart rate, SBP, TC, FG [with or without impaired fasting glucose (IFG: defined as a FG level ≥ 100.8 and < 126.0 mg/dL)], and folate at baseline, as well as the time-averaged SBP during the follow-up period.

### Comparisons with previous studies

Our results indicate that the traditional assumption that diabetes precedes arterial stiffness might need to be reconsidered. While some previous cross-sectional studies [13–15] have reported a positive association between baPWV and the prevalence of diabetes, the prospective association between diabetes and arterial stiffness is however, still inconclusive. de Oliveira Alvim et al. [26] found no significant difference in PWV progression after a 5-year follow-up in a subset of diabetics compared to non-diabetics. In contrast, Ferreira et al. [27] reported that better glycemic control, together with reductions in blood pressure and heart rate, was inversely associated with PWV changes. These results show that the association between arterial stiffness and diabetes is likely bidirectional. Diabetes might be in part a consequence of vascular impairment [28].

Our study findings are supported by previous studies. First, in a study on patients with untreated essential hypertension [29], those with higher pulse pressure (PP) exhibited impaired insulin secretion, increased post-challenge glucose concentrations and greater glucose spikes (PGS) during 75 g oral glucose tolerance testing. Our current study also found that there was a significant positive relationship of time-averaged PP with new-onset diabetes. Moreover, the positive association between baPWV and new-onset diabetes was independent of baseline PP and time-averaged PP during the treatment period. These results suggested that baPWV may be a more accurate marker of arterial stiffness. Second, baPWV has been shown to be associated with endothelial dysfunction [30], hypertension [10], inflammation [31], triglyceride glucose (TyG) index [32], left ventricular hypertrophy (LVH) [12] and lower muscle tissue [33]. Previous studies have found that endothelial dysfunction [34], hypertension [35], inflammation [36], TyG index [37], and LVH [28, 38, 39] precede incident diabetes. Moreover, patients with type 2 diabetes and visceral fat accumulation usually had lower muscle quality [40]. Third, previous clinical trials in diabetic patients indicated that the control of hypertension and hypercholesterolemia is more effective than glucose-lowering therapy in reducing cardiovascular events [41]. Fourth, angiotensin II receptor blocker (ARB) was found to inhibit the progression of arterial stiffness independent of blood pressure reduction [42]. Accordingly, ARB treatment significantly reduced the incidence of diabetes in previous studies [43, 44]. Our results provide further evidence for the assumption that diabetes may be partly a disease of vascular origin. However, further studies are needed to verify this hypothesis.

### Possible mechanisms

The exact mechanisms underlying the relationship of baPWV with new-onset diabetes remain to be elucidated. Some previously proposed potential mechanisms are outlined here. The propagation of increased pressure and flow pulsations to the pancreatic bed may lead to pancreatic dysfunction [18]. At the same time, arterial stiffness leads to increased arterial pulse pressure and pulsatile shear, resulting in endothelial dysfunction and metabolic dysregulation [30]. Endothelial dysfunction and impaired endothelium-dependent vasodilation may exacerbate insulin resistance by limiting the delivery of glucose to key target tissues [34]. More studies are needed to confirm our findings, and to further investigating the underlying mechanisms involved in this association.

## Limitations

Our study has some limitations. First, arterial stiffness might represent the result of a number of risk factors, while the regression models were adjusted for a broad array of covariates, residual confounding from unmeasured factors cannot be excluded. Second, our present study was conducted in hypertensive participants, the generalizability of the results to adults without hypertension remains to be examined. Third, we did not measure glycosylated hemoglobin A1c or perform glucose tolerance tests. However, our definition of diabetes was similar to that of previous randomized trials [45, 46] or observational studies [25, 47, 48]. In addition, a glucose tolerance test is difficult to perform in practice, particularly in rural China. Finally, we did not have direct assessments of endothelial and β-cell function and insulin levels, which would have helped our understanding of the underlying pathophysiology and temporal sequence between baPWV and diabetes. Overall, our study served as hypothesis-generating; all findings need to be further investigated and confirmed in future studies.

## Conclusions

In this sample of hypertensive patients, we found a significant positive association between baseline baPWV and the risk of new-onset diabetes during follow-up. If further confirmed, baPWV measurements along with other known risk factors could further help identify hypertensive patients at high-risk of developing diabetes. By targeting these high-risk patients and implementing early intensive multiple vascular risk factor control, we may help reduce their future diabetes risk.

## Supplementary information

**Additional file 1.** **Table S1.** Baseline characteristics of the study population by brachial–ankle pulse wave velocity (baPWV) quartiles. **Table S2.** Association between baseline brachial–ankle pulse wave velocity (baPWV) and new-onset diabetes during follow-up. **Table S3.** Association between baseline brachial–ankle pulse wave velocity (baPWV) and physician-diagnosed diabetes or use of glucose-lowering drugs during follow-up. **Table S4.** Association between baseline brachial–ankle pulse wave velocity (baPWV) and the change in fasting glucose among subjects without physician-diagnosed diabetes, or use of glucose-lowering drugs during follow-up. **Table S5.** Concomitant use of medications during the treatment period by the quartiles of baseline brachial–ankle pulse wave velocity (baPWV). **Table S6.** Association between baseline brachial–ankle pulse wave velocity (baPWV) and new-onset diabetes during follow up, with further adjustment for the use of calcium channel blockers or diuretics during the treatment period. **Table S7.** Association between baseline brachial–ankle pulse wave velocity (baPWV) and new-onset diabetes during follow-up in participants without the use of diuretics during the treatment period. **Table S8.** Association between baseline brachial–ankle pulse wave velocity (baPWV) and new-onset diabetes during follow-up, with further adjustment for the study drug (enalapril or enalapril-folic acid) compliance during the trial. **Table S9.** Association between baseline brachial–ankle pulse wave velocity (baPWV) and new-onset diabetes during follow-up, with further adjustment for alcohol consumption, family history of diabetes, physical activity and socioeconomic status. **Table S10. **Association between baseline brachial–ankle pulse wave velocity (baPWV) and new-onset diabetes during follow-up, with further adjustment for change in BMI (calculated as BMI at the exit visit minus that at baseline). **Table S11.** Association between baseline pulse pressure (PP) (both baseline PP and time-averaged PP during the treatment period) and new-onset diabetes during follow-up. **Table S12.** Association between baseline brachial–ankle pulse wave velocity (baPWV) and new-onset diabetes during follow up, with adjustment for pulse pressure (PP) with or without systolic blood pressure (SBP). **Figure S1.** Flow chart of the study participants. **Figure S2.** Association between baseline brachial–ankle pulse wave velocity (baPWV) and physician-diagnosed diabetes or use of glucose-lowering drugs during follow-up. **Figure S3.** Association between baseline brachial–ankle pulse wave velocity (baPWV) and the change in fasting glucose (fasting glucose at the exit visit minus that at baseline) among subjects without physician-diagnosed diabetes, or use of glucose-lowering drugs during follow-up.

## Data Availability

The data and study materials that support the findings of this study will be available from the corresponding authors upon request, after the request is submitted and formally reviewed and approved by the Ethics Committee of the Institute of Biomedicine, Anhui Medical University.

## Abbreviations

ABI: ankle–brachial index
baPWV: brachial–ankle pulse wave velocity
BP: blood pressure
CCB: calcium channel blockers
CVD: cardiovascular disease
CSPPT: China Stroke Primary Prevention Trial
DBP: diastolic blood pressure
FG: fasting glucose
HDL-C: high-density lipoprotein
LVH: left ventricular hypertrophy
*MTHFR*: methylenetetrahydrofolate reductase
PP: pulse pressure
PAD: peripheral artery occlusive disease
SBP: systolic blood pressure
