# Supplementary material for: Positive association between baseline brachial–ankle pulse wave velocity and the risk of new-onset diabetes in hypertensive patients
Source: Cardiovasc Diabetol. 2019 Aug 28;18:111. doi: 10.1186/s12933-019-0915-0 (PMC6714437; doi:10.1186/s12933-019-0915-0)
Supplement: Supplementary file 1 — Additional file 1. Table S1. Baseline characteristics of the study population by brachial–ankle pulse wave velocity (baPWV) quartiles. Table S2. Association between baseline brachial–ankle pulse wave velocity (baPWV) and new-onset diabetes during follow-up. Table S3. Association between baseline brachial–ankle pulse wave velocity (baPWV) and physician-diagnosed diabetes or use of glucose-lowering drugs during follow-up. Table S4. Association between baseline brachial–ankle pulse wave velocity (baPWV) and the change in fasting glucose among subjects without physician-diagnosed diabetes, or use of glucose-lowering drugs during follow-up. Table S5. Concomitant use of medications during the treatment period by the quartiles of baseline brachial–ankle pulse wave velocity (baPWV). Table S6. Association between baseline brachial–ankle pulse wave velocity (baPWV) and new-onset diabetes during follow up, with further adjustment for the use of calcium channel blockers or diuretics during the treatment period. Table S7. Association between baseline brachial–ankle pulse wave velocity (baPWV) and new-onset diabetes during follow-up in participants without the use of diuretics during the treatment period. Table S8. Association between baseline brachial–ankle pulse wave velocity (baPWV) and new-onset diabetes during follow-up, with further adjustment for the study drug (enalapril or enalapril-folic acid) compliance during the trial. Table S9. Association between baseline brachial–ankle pulse wave velocity (baPWV) and new-onset diabetes during follow-up, with further adjustment for alcohol consumption, family history of diabetes, physical activity and socioeconomic status. Table S10. Association between baseline brachial–ankle pulse wave velocity (baPWV) and new-onset diabetes during follow-up, with further adjustment for change in BMI (calculated as BMI at the exit visit minus that at baseline). Table S11. Association between baseline pulse pressure (PP) (both baseline PP and time [file 12933_2019_915_MOESM1_ESM.docx]

**Positive association between baseline** **brachial-ankle pulse wave velocity and the risk of new-onset diabetes in hypertensive patients**

20702 participants in CSPPT

3532 participants with brachial-ankle pulse wave velocity (baPWV) data at baseline

Excluded n=1103

ABI<0.90, n=221

With diabetes at baseline, n=411;

Missing glucose at baseline or exit visit and without physician-diagnosed diabetes or use of glucose-lowering drugs during the follow-up, n=471

Excluded n=5724

With diabetes at baselnie, n=2649

Missing glucose at baseline or exit visit except physician-diagnosed diabetes or use of glucose-lowering drugs during the follow-up , n=3075

2429 participants in final analysis

No new-onset diabetes n=2142 (88.2%)

New-onset diabetes n=287 (11.8%)

**Figure S1. Flow chart of the study participants**

**
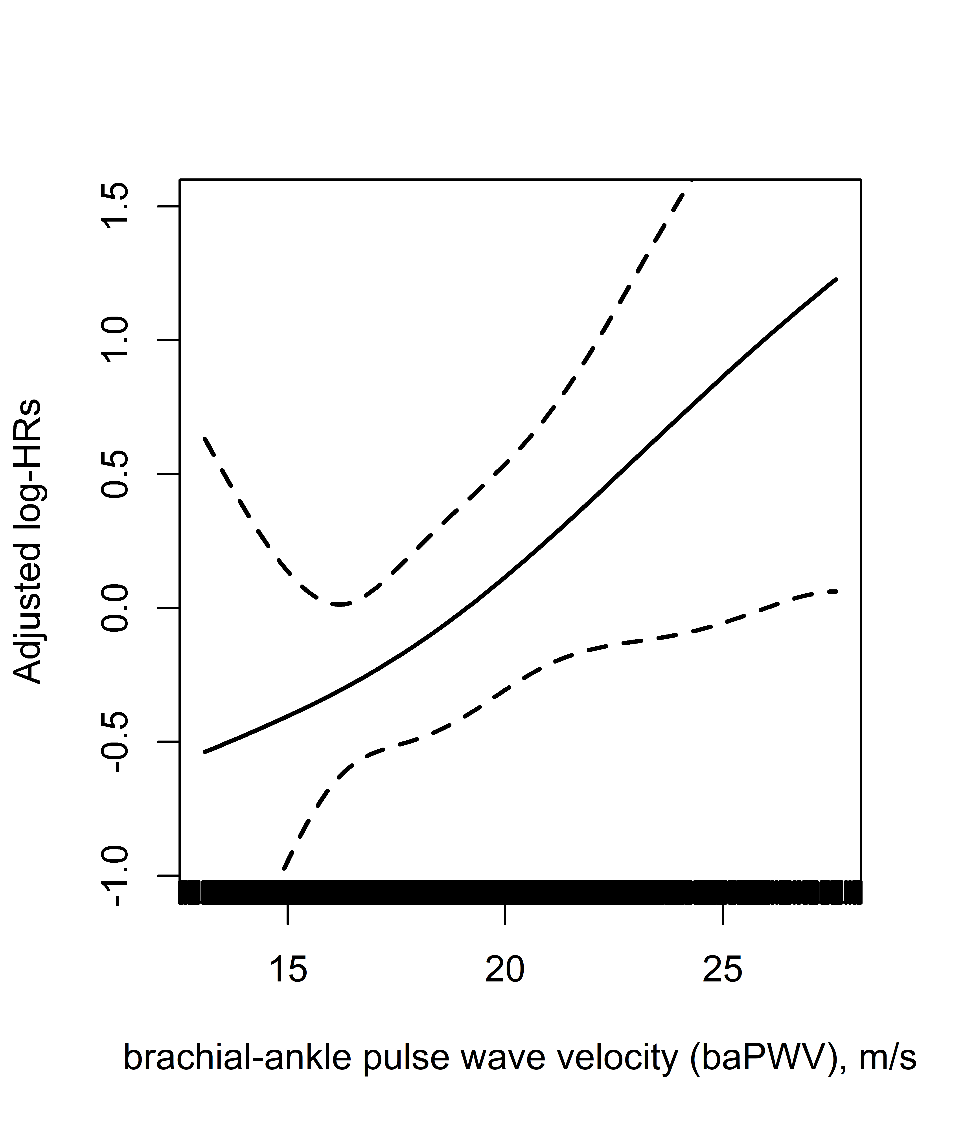
**

**Figure S2. Association between baseline brachial-ankle pulse wave velocity (baPWV) and physician-diagnosed diabetes or use of glucose-lowering drugs during follow-up**

*Adjusted for age, sex, study center, study treatment group, body mass index (BMI), heart rate, smoking, systolic blood pressure (SBP), fasting glucose (FG), total cholesterol (TC), creatinine, and folate at baseline, as well as time-averaged SBP during the treatment period.


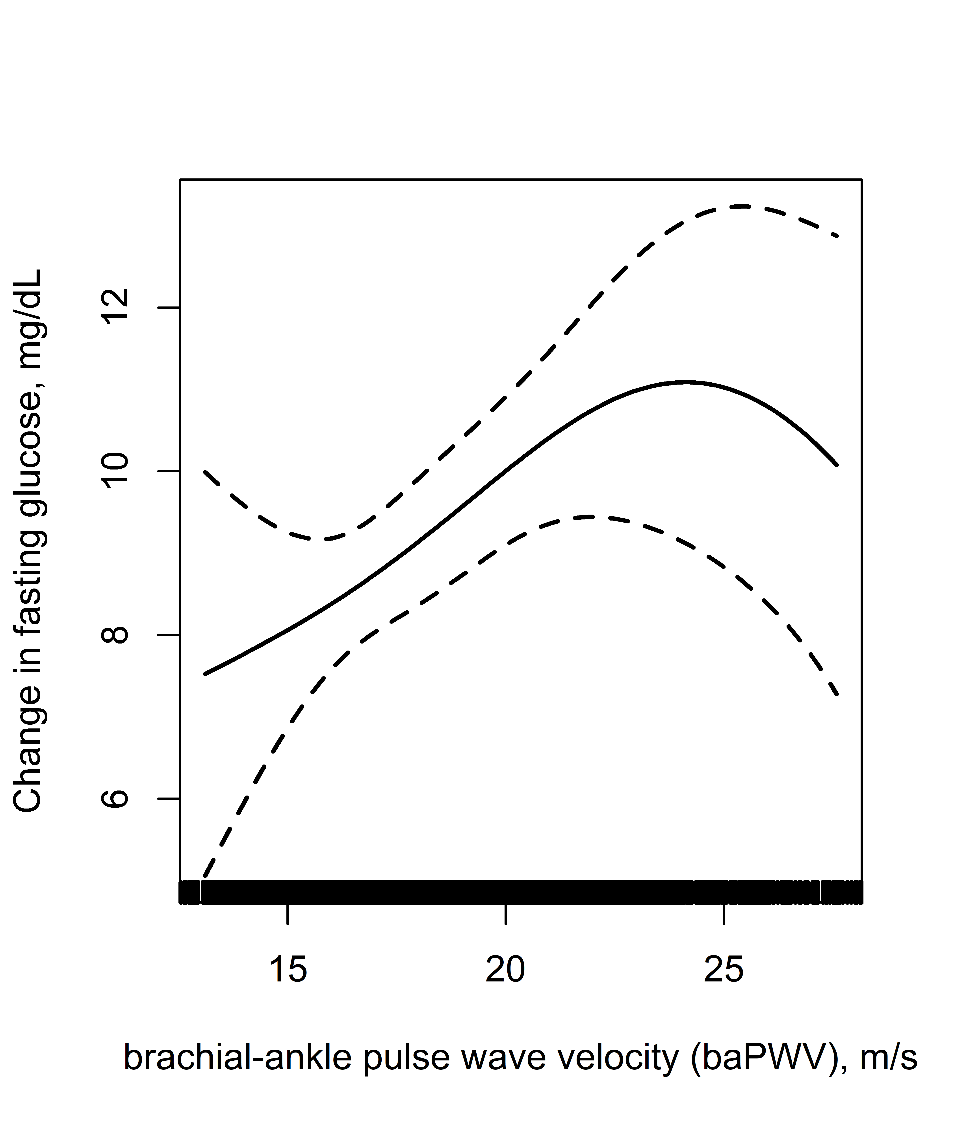


**Figure S3. Association between baseline brachial-ankle pulse wave velocity (baPWV) and the change in fasting glucose (fasting glucose at the exit visit minus that at baseline) among subjects without physician-diagnosed diabetes, or use of glucose-lowering drugs during follow-up**

*Adjusted for age, sex, study center, study treatment group, body mass index (BMI), heart rate, smoking, systolic blood pressure (SBP), fasting glucose (FG), total cholesterol (TC), creatinine, and folate at baseline, as well as time-averaged SBP during the treatment period.

| Variables, No. (%) | baPWV, m/s | | | |  |
| --- | --- | --- | --- | --- | --- |
|  | Quartile 1  (<15.9) | Quartile 2  (15.9-<17.9) | Quartile 3  (17.9-<20.7) | Quartile 4  (≥20.7) | *P* value |
| N | 607 | 605 | 606 | 611 |  |
| **Alcohol consumption** |  |  |  |  | 0.589 |
| Never | 397 (65.5) | 414 (68.4) | 430 (71.0) | 415 (68.0) |  |
| Ever | 47 (7.8) | 39 (6.4) | 36 (5.9) | 39 (6.4) |  |
| Current | 162 (26.7) | 152 (25.1) | 140 (23.1) | 156 (25.6) |  |
| **Family history of diabetes** |  |  |  |  | 0.470 |
| Yes | 27 (4.5) | 22 (3.6) | 17 (2.8) | 25 (4.1) |  |
| No | 579 (95.5) | 583 (96.4) | 587 (97.2) | 584 (95.9) |  |
| **Physical activity** |  |  |  |  | <0.001 |
| Low | 191 (31.5) | 197 (32.6) | 225 (37.1) | 268 (43.9) |  |
| Moderate | 241 (39.7) | 249 (41.2) | 236 (38.9) | 227 (37.2) |  |
| High | 175 (28.8) | 159 (26.3) | 145 (23.9) | 115 (18.9) |  |
| **Socioeconomic status** |  |  |  |  | 0.735 |
| Poor | 67 (11.0) | 74 (12.2) | 77 (12.7) | 73 (11.9) |  |
| Medium | 461 (75.9) | 465 (76.9) | 469 (77.4) | 470 (76.9) |  |
| Good | 79 (13.0) | 66 (10.9) | 60 (9.9) | 68 (11.1) |  |

**Table S1. Baseline characteristics of the study population by brachial-ankle pulse wave velocity (baPWV) quartiles^*^**

*Variables are presented as n (%)

**Table S2. Association between baseline brachial-ankle pulse wave velocity (baPWV) and new-onset diabetes during follow-up**

| baPWV, m/s | N | No. of events (%) | Crude Model | | Adjusted Model^*^ | | |
| --- | --- | --- | --- | --- | --- | --- | --- |
|  |  |  | OR (95%CI) | *P* value | OR (95%CI) | *P* value | |
| Continuous, Per SD increment | 2429 | 287 (11.8) | 1.26 (1.13, 1.42) | <0.001 | 1.33 (1.13, 1.56) | | <0.001 |
| Quartiles |  |  |  |  |  | |  |
| Q1(<15.9) | 607 | 46 (7.6) | *ref.* |  | *ref.* | |  |
| Q2(15.9-<17.9) | 605 | 68 (11.2) | 1.54 (1.04, 2.29) | 0.030 | 1.63 (1.06, 2.49) | | 0.025 |
| Q3(17.9-<20.7) | 606 | 76 (12.5) | 1.75 (1.19, 2.57) | 0.004 | 1.87 (1.20, 2.91) | | 0.006 |
| Q4(≥20.7) | 611 | 97 (15.9) | 2.30 (1.59, 3.33) | <0.001 | 2.48 (1.53, 4.03) | | <0.001 |
| *P* for trend |  |  | <0.001 |  | <0.001 | |  |
| Categories |  |  |  |  |  | |  |
| Q1(<15.9) | 607 | 46 (7.6) | *ref.* |  | *ref.* | |  |
| Q2-4(≥15.9) | 1822 | 241 (13.2) | 1.86 (1.34, 2.59) | <0.001 | 1.80 (1.22, 2.65) | | 0.003 |

*Adjusted for age, sex, study center, study treatment group, body mass index (BMI), heart rate, smoking, systolic blood pressure (SBP), fasting glucose (FG), total cholesterol (TC), creatinine, and folate at baseline, as well as time-averaged SBP during the treatment period.

**Table S3. Association between baseline brachial-ankle pulse wave velocity (baPWV) and physician-diagnosed diabetes or use of glucose-lowering drugs during follow-up**

| baPWV, m/s | N | No. of events (%) | Crude Model | | Adjusted Model* | |
| --- | --- | --- | --- | --- | --- | --- |
|  |  |  | HR (95%CI) | *P* value | HR (95%CI) | *P* value |
| Continuous, Per SD increment | 2429 | 30 (1.2) | 1.25 (0.91, 1.72) | 0.162 | 1.61 (1.05, 2.47) | 0.031 |
| Quartiles |  |  |  |  |  |  |
| Q1(<15.9) | 607 | 6 (1.0) | *ref.* |  | *ref.* |  |
| Q2(15.9-17.9) | 605 | 3 (0.5) | 0.48 (0.12, 1.92) | 0.300 | 0.56 (0.14, 2.35) | 0.431 |
| Q3(17.9-20.7) | 606 | 11 (1.8) | 1.70 (0.63, 4.61) | 0.294 | 1.81 (0.61, 5.41) | 0.288 |
| Q4(≥20.7) | 611 | 10 (1.6) | 1.51 (0.55, 4.15) | 0.429 | 2.25 (0.65, 7.74) | 0.198 |
| *P* for trend |  |  | 0.157 |  | 0.086 |  |
| Categories |  |  |  |  |  |  |
| Q1-2(<17.9) | 1212 | 9 (0.7) | *ref.* |  | *ref.* |  |
| Q3-4(≥17.9) | 1217 | 21 (1.7) | 2.18 (1.00, 4.77) | 0.051 | 2.50 (1.02, 6.10) | 0.044 |

*Adjusted for age, sex, study center, study treatment group, body mass index (BMI), heart rate, smoking, systolic blood pressure (SBP), fasting glucose (FG), total cholesterol (TC), creatinine, and folate at baseline, as well as time-averaged SBP during the treatment period.

**Table S4. Association between baseline brachial-ankle pulse wave velocity (baPWV) and the change in fasting glucose among subjects without physician-diagnosed diabetes, or use of glucose-lowering drugs during follow-up**

| baPWV, m/s | N | Mean (SD) | Crude Model | | Adjusted Model* | |
| --- | --- | --- | --- | --- | --- | --- |
|  |  |  | β (95%CI) | *P* value | β (95%CI) | *P* value |
| Continuous, Per SD increment | 2399 | 9.2 (20.3) | 0.59 (-0.22, 1.40) | 0.156 | 0.63 (-0.37, 1.64) | 0.217 |
| Quartiles |  |  |  |  |  |  |
| Q1(<15.9) | 598 | 7.8 (19.4) | *ref.* |  | *ref.* |  |
| Q2(15.9-17.9) | 601 | 9.5 (18.8) | 1.70 (-0.59, 4.00) | 0.145 | 1.72 (-0.56, 3.99) | 0.139 |
| Q3(17.9-20.7) | 599 | 9.5 (21.4) | 1.69 (-0.61, 3.98) | 0.150 | 1.51 (-0.92, 3.94) | 0.225 |
| Q4(≥20.7) | 601 | 10.0 (21.4) | 2.22 (-0.08, 4.51) | 0.059 | 2.89 (0.12, 5.66) | 0.041 |
| *P* for trend |  |  | 0.074 |  | 0.067 |  |
| Categories |  |  |  |  |  |  |
| Q1(<15.9) | 598 | 7.8 (19.4) | *ref.* |  | *ref.* |  |
| Q2-4 (≥15.9) | 1801 | 9.7 (20.5) | 1.87 (0.00, 3.74) | 0.051 | 1.77 (-0.28, 3.83) | 0.091 |

*Adjusted for age, sex, study center, study treatment group, body mass index (BMI), heart rate, smoking, systolic blood pressure (SBP), fasting glucose (FG), total cholesterol (TC), creatinine, and folate at baseline, as well as time-averaged SBP during the treatment period.

**Table S5. Concomitant use of medications** **during the treatment period by the quartiles of baseline brachial-ankle pulse wave velocity (baPWV)^*^**

| Medication, No. (%) | baPWV, m/s | | | |  |
| --- | --- | --- | --- | --- | --- |
|  | Quartile 1  (<15.9) | Quartile 2  (15.9-<17.9) | Quartile 3  (17.9-<20.7) | Quartile 4  (≥20.7) | *P* value |
| N | 607 | 605 | 606 | 611 |  |
| Anti-hypertension drugs |  |  |  |  |  |
| Calcium channel blockers | 436 (71.8) | 504 (83.3) | 521 (86.0) | 545 (89.2) | <0.001 |
| Diuretics | 274 (45.1) | 325 (53.7) | 349 (57.6) | 410 (67.1) | <0.001 |
| beta blockers | 1 (0.2) | 3 (0.5) | 2 (0.3) | 0 (0.0) | 0.338 |
| Lipid-lowering drugs | 1 (0.2) | 1 (0.2) | 3 (0.5) | 1 (0.2) | 0.569 |
| Antiplatelet drugs | 5 (0.8) | 4 (0.7) | 5 (0.8) | 3 (0.5) | 0.881 |

* Concomitant use of medications was defined as 180 or more cumulative days of taking the drug of interest.

| baPWV, m/s | N | No. of events (%) | Adjusted Model^*^ | |
| --- | --- | --- | --- | --- |
|  |  |  | OR (95%CI) | *P* value |
| Continuous, Per SD increment | 2429 | 287 (11.8) | 1.33 (1.13, 1.56) | <0.001 |
| Quartiles |  |  |  |  |
| Q1(<15.9) | 607 | 46 (7.6) | *Ref* |  |
| Q2(15.9-<17.9) | 605 | 68 (11.2) | 1.63 (1.06, 2.49) | 0.025 |
| Q3(17.9-<20.7) | 606 | 76 (12.5) | 1.87 (1.20, 2.91) | 0.005 |
| Q4(≥20.7) | 611 | 97 (15.9) | 2.49 (1.53, 4.05) | <0.001 |
| *P* for trend |  |  | <0.001 |  |
| Categories |  |  |  |  |
| Q1(<15.9) | 607 | 46 (7.6) | *ref.* |  |
| Q2-4(≥15.9) | 1822 | 241 (13.2) | 1.80 (1.22, 2.65) | 0.003 |

**Table S6. Association between baseline brachial-ankle pulse wave velocity (baPWV) and new-onset diabetes during follow up, with further adjustment for the use of calcium channel blockers or diuretics during the treatment period**

*Adjusted for age, sex, study center, study treatment group, body mass index (BMI), heart rate, smoking, systolic blood pressure (SBP), fasting glucose (FG), total cholesterol (TC), creatinine, and folate at baseline, as well as time-averaged SBP, and the use of calcium channel blockers (CCB) or diuretics during the treatment period.

**Table S7.** **Association between baseline brachial-ankle pulse wave velocity (baPWV) and new-onset diabetes during follow-up in participants without the use of diuretics during the treatment period**

| baPWV, m/s | N | No. of events (%) | Crude Model | | Adjusted Model* | |
| --- | --- | --- | --- | --- | --- | --- |
|  |  |  | OR (95%CI) | *P* value | OR (95%CI) | *P* value |
| Continuous, Per SD increment | 1071 | 116 (10.8) | 1.31 (1.07, 1.59) | 0.009 | 1.32 (1.00, 1.74) | 0.050 |
| Quartiles |  |  |  |  |  |  |
| Q1(<15.6) | 268 | 20 (7.5) | *ref.* |  | *ref.* |  |
| Q2(15.6-17.4) | 267 | 27 (10.1) | 1.39 (0.76, 2.55) | 0.281 | 1.42 (0.74, 2.72) | 0.296 |
| Q3(17.4-19.9) | 268 | 27 (10.1) | 1.39 (0.76, 2.54) | 0.287 | 1.27 (0.63, 2.57) | 0.503 |
| Q4(≥19.9) | 268 | 42 (15.7) | 2.30 (1.31, 4.04) | 0.004 | 2.24 (1.07, 4.68) | 0.032 |
| *P* for trend |  |  | 0.004 |  | 0.048 |  |
| Categories |  |  |  |  |  |  |
| Q1(<15.6) | 268 | 20 (7.5) | *ref.* |  | *ref.* |  |
| Q2-4(≥15.6) | 803 | 96 (12.0) | 1.68 (1.02, 2.78) | 0.042 | 1.46 (0.81, 2.65) | 0.207 |

*Adjusted for age, sex, study center, study treatment group, body mass index (BMI), heart rate, smoking, systolic blood pressure (SBP), fasting glucose (FG), total cholesterol (TC), creatinine and folate at baseline, as well as time-averaged SBP during the treatment period.

**Table S8. Association between baseline brachial-ankle pulse wave velocity (baPWV) and new-onset diabetes during follow-up, with further adjustment for the study drug (enalapril or enalapril-folic acid) compliance during the trial.**

| baPWV, m/s | N | No. of events (%) | Crude Model | | Adjusted Model^*^ | | |
| --- | --- | --- | --- | --- | --- | --- | --- |
|  |  |  | OR (95%CI) | *P* value | OR (95%CI) | *P* value | |
| Continuous, Per SD increment | 2429 | 287 (11.8) | 1.26 (1.13, 1.42) | <0.001 | 1.33 (1.14, 1.56) | | <0.001 |
| Quartiles |  |  |  |  |  | |  |
| Q1(<15.9) | 607 | 46 (7.6) | *ref.* |  | *ref.* | |  |
| Q2(15.9-<17.9) | 605 | 68 (11.2) | 1.54 (1.04, 2.29) | 0.030 | 1.64 (1.07, 2.51) | | 0.024 |
| Q3(17.9-<20.7) | 606 | 76 (12.5) | 1.75 (1.19, 2.57) | 0.004 | 1.88 (1.21, 2.92) | | 0.005 |
| Q4(≥20.7) | 611 | 97 (15.9) | 2.30 (1.59, 3.33) | <0.001 | 2.52 (1.55, 4.10) | | <0.001 |
| *P* for trend |  |  | <0.001 |  | <0.001 | |  |
| Categories |  |  |  |  |  | |  |
| Q1(<15.9) | 607 | 46 (7.6) | *ref.* |  | *ref.* | |  |
| Q2-4(≥15.9) | 1822 | 241 (13.2) | 1.86 (1.34, 2.59) | <0.001 | 1.81 (1.23, 2.66) | | 0.003 |

*Adjusted for age, sex, study center, study treatment group, body mass index (BMI), heart rate, smoking, systolic blood pressure (SBP), fasting glucose (FG), total cholesterol (TC), creatinine, and folate at baseline, as well as time-averaged SBP and the study drug (enalapril or enalapril-folic acid) compliance during the trial.

**Table S9. Association between baseline brachial-ankle pulse wave velocity (baPWV) and new-onset diabetes during follow-up, with further adjustment for alcohol consumption, family history of diabetes, physical activity and socioeconomic status.**

| baPWV, m/s | N | No. of events (%) | Crude Model | | Adjusted Model^*^ | | |
| --- | --- | --- | --- | --- | --- | --- | --- |
|  |  |  | OR (95%CI) | *P* value | OR (95%CI) | *P* value | |
| Continuous, Per SD increment | 2429 | 287 (11.8) | 1.26 (1.13, 1.42) | <0.001 | 1.33 (1.13, 1.56) | | <0.001 |
| Quartiles |  |  |  |  |  | |  |
| Q1(<15.9) | 607 | 46 (7.6) | *ref.* |  | *ref.* | |  |
| Q2(15.9-<17.9) | 605 | 68 (11.2) | 1.54 (1.04, 2.29) | 0.030 | 1.63 (1.06, 2.50) | | 0.025 |
| Q3(17.9-<20.7) | 606 | 76 (12.5) | 1.75 (1.19, 2.57) | 0.004 | 1.88 (1.21, 2.94) | | 0.005 |
| Q4(≥20.7) | 611 | 97 (15.9) | 2.30 (1.59, 3.33) | <0.001 | 2.45 (1.50, 4.00) | | <0.001 |
| *P* for trend |  |  | <0.001 |  | <0.001 | |  |
| Categories |  |  |  |  |  | |  |
| Q1(<15.9) | 607 | 46 (7.6) | *ref.* |  | *ref.* | |  |
| Q2-4(≥15.9) | 1822 | 241 (13.2) | 1.86 (1.34, 2.59) | <0.001 | 1.80 (1.22, 2.66) | | 0.003 |

*Adjusted for age, sex, study center, study treatment group, body mass index (BMI), heart rate, smoking, alcohol consumption, family history of diabetes, physical activity, socioeconomic status, systolic blood pressure (SBP), fasting glucose (FG), total cholesterol (TC), creatinine, and folate at baseline, as well as time-averaged SBP during the treatment period.

**Table S10. Association between baseline brachial-ankle pulse wave velocity (baPWV) and new-onset diabetes during follow-up, with further adjustment for change in BMI (calculated as BMI at the exit visit minus that at baseline).**

| baPWV, m/s | N | No. of events (%) | Crude Model | | Adjusted Model^*^ | | |
| --- | --- | --- | --- | --- | --- | --- | --- |
|  |  |  | OR (95%CI) | *P* value | OR (95%CI) | *P* value | |
| Continuous, Per SD increment | 2429 | 287 (11.8) | 1.26 (1.13, 1.42) | <0.001 | 1.33 (1.14, 1.57) | | <0.001 |
| Quartiles |  |  |  |  |  | |  |
| Q1(<15.9) | 607 | 46 (7.6) | *ref.* |  | *ref.* | |  |
| Q2(15.9-<17.9) | 605 | 68 (11.2) | 1.54 (1.04, 2.29) | 0.030 | 1.65 (1.07, 2.53) | | 0.022 |
| Q3(17.9-<20.7) | 606 | 76 (12.5) | 1.75 (1.19, 2.57) | 0.004 | 1.93 (1.23, 3.01) | | 0.004 |
| Q4(≥20.7) | 611 | 97 (15.9) | 2.30 (1.59, 3.33) | <0.001 | 2.52 (1.54, 4.12) | | <0.001 |
| *P* for trend |  |  | <0.001 |  | <0.001 | |  |
| Categories |  |  |  |  |  | |  |
| Q1(<15.9) | 607 | 46 (7.6) | *ref.* |  | *ref.* | |  |
| Q2-4(≥15.9) | 1822 | 241 (13.2) | 1.86 (1.34, 2.59) | <0.001 | 1.83 (1.24, 2.70) | | 0.002 |

*Adjusted for age, sex, study center, study treatment group, body mass index (BMI), change in BMI, heart rate, smoking, alcohol consumption, family history of diabetes, physical activity and socioeconomic status systolic blood pressure (SBP), fasting glucose (FG), total cholesterol (TC), creatinine, and folate at baseline, as well as time-averaged SBP during the treatment period.

**Table S11. Association between baseline pulse pressure (PP) (both baseline PP and time-averaged PP during the treatment period) and new-onset diabetes during follow-up**

| PP, mmHg | N | No. of events (%) | Crude Model | | Adjusted Model* | | |
| --- | --- | --- | --- | --- | --- | --- | --- |
|  |  |  | OR (95%CI) | *P* value | OR (95%CI) | *P* value | |
| **PP at baseline** |  |  |  |  |  | |  |
| Quartiles |  |  |  |  |  | |  |
| Q1(<59) | 606 | 68 (11.2) | *ref.* |  | *ref.* | |  |
| Q2(59-<71) | 608 | 67 (11.0) | 0.98 (0.69, 1.40) | 0.911 | 0.82 (0.56, 1.21) | | 0.321 |
| Q3(71-<82) | 607 | 76 (12.5) | 1.13 (0.80, 1.60) | 0.484 | 0.85 (0.57, 1.27) | | 0.439 |
| Q4(≥82) | 608 | 76 (12.5) | 1.13 (0.80, 1.60) | 0.491 | 0.83 (0.52, 1.30) | | 0.407 |
| **Time-averaged PP during the treatment period** | | |  |  |  | |  |
| Quartiles |  |  |  |  |  | |  |
| Q1(<48) | 607 | 51 (8.4) | *ref.* |  | *ref.* | |  |
| Q2(48-<55) | 607 | 73 (12.0) | 1.49 (1.02, 2.17) | 0.038 | 1.27 (0.84, 1.91) | | 0.256 |
| Q3(55-<62) | 607 | 81 (13.3) | 1.68 (1.16, 2.43) | 0.006 | 1.56 (1.02, 2.39) | | 0.040 |
| Q4(≥62) | 608 | 82 (13.5) | 1.70 (1.17, 2.46) | 0.005 | 1.52 (0.94, 2.44) | | 0.084 |
| Categories |  |  |  |  |  | |  |
| Q1(<48) | 607 | 51 (8.4) | *ref.* |  | *ref.* | |  |
| Q2(48-<55) | 607 | 73 (12.0) | 1.49 (1.02, 2.17) | 0.038 | 1.27 (0.84, 1.91) | | 0.256 |
| Q3-4(≥55) | 1215 | 163 (13.4) | 1.69 (1.21, 2.35) | 0.002 | 1.55 (1.03, 2.33) | | 0.036 |

*Adjusted for age, sex, study center, study treatment group, body mass index (BMI), heart rate, smoking, fasting glucose (FG), total cholesterol (TC), creatinine, folate and pulse pressure (PP) at baseline, as well as time-averaged PP during the treatment period.

**Table S12.** **Association between baseline brachial-ankle pulse wave velocity (baPWV) and new-onset diabetes during follow up, with adjustment for pulse pressure (PP) with or without systolic blood pressure (SBP)**

| baPWV, m/s | N | No. of events (%) | Model 1 | | Model 2 | | |
| --- | --- | --- | --- | --- | --- | --- | --- |
|  |  |  | OR (95%CI) | *P* value | OR (95%CI) | *P* value | |
| Continuous, Per SD increment | 2429 | 287 (11.8) | 1.35 (1.16, 1.57) | <0.001 | 1.30 (1.11, 1.61) | | 0.004 |
| Quartiles |  |  |  |  |  | |  |
| Q1(<15.9) | 607 | 46 (7.6) | *ref.* |  | *ref.* | |  |
| Q2(15.9-<17.9) | 605 | 68 (11.2) | 1.67 (1.09, 2.55) | 0.018 | 1.61 (1.05, 2.47) | | 0.028 |
| Q3(17.9-<20.7) | 606 | 76 (12.5) | 1.95 (1.26, 3.02) | 0.003 | 1.86 (1.19, 2.89) | | 0.006 |
| Q4(≥20.7) | 611 | 97 (15.9) | 2.63 (1.65, 4.19) | <0.001 | 2.39 (1.46, 3.91) | | <0.001 |
| *P* for trend |  |  | <0.001 |  | <0.001 | |  |
| Categories |  |  |  |  |  | |  |
| Q1(<15.9) | 607 | 46 (7.6) | *ref.* |  | *ref.* | |  |
| Q2-4(≥15.9) | 1822 | 241 (13.2) | 1.91 (1.30, 2.79) | <0.001 | 1.77 (1.20, 2.61) | | 0.004 |

Model 1: Adjusted for age, sex, study center, study treatment group, body mass index (BMI), heart rate, smoking, pulse pressure (PP), fasting glucose (FG), total cholesterol (TC), creatinine, and folate at baseline, as well as time-averaged PP during the treatment period.

Model 2: Adjusted for all variables in model 1 plus SBP at baseline and time-averaged SBP during the treatment period.
